# The complete chloroplast genome of *Acer Pubipetiolatum* var. *pingpienense* (Sapindaceae)

**DOI:** 10.1080/23802359.2022.2107443

**Published:** 2022-08-08

**Authors:** Detuan Liu, Suiyun Chen, Weibang Sun

**Affiliations:** a School of Life Sciences, Yunnan University, Kunming, China; b Yunnan Key Laboratory for Integrative Conservation of Plant Species with Extremely Small Populations, Kunming Institute of Botany, Chinese Academy of Sciences, Kunming, China; c School of Ecology and Environmental Science, Yunnan University, Kunming, China

**Keywords:** chloroplast, *Acer*, structure, phylogeny

## Abstract

The genus *Acer* is widespread throughout the northern temperate zone, and many species within the genus are of ecological and economical importance. Here we report the newly sequenced chloroplast genome of *Acer pubipetiolatum* var. *pingpienense*. This chloroplast genome has a total length of 156,730 bp, and contains a pair of inverted repeats (IRs, 26,743 bp), a large single-copy (LSC) region of 71,582 bp and a small single-copy (SSC) region of 18,092 bp. Phylogenetic analysis suggests that *A. pubipetiolatum* var. *pingpienense* is closely related to *A. laevigatum*, and both fall into Section *Palmata*. The complete *A. pubipetiolatum* var. *pingpienense* chloroplast genome will provide an important genetic resource for future research into the conservation and evolution of this genus. Our findings also suggest that further research is necessary to elucidate the phylogenetic relationships between plant species within this genus.

The chloroplast (cp) genome, which is independent of that of the cell nucleus, is a maternally inherited genetic unit, and has highly conserved gene size and structure (Palmer 1991). Knowledge of the chloroplast genome has applications in the fields of molecular evolution, species identification, molecular breeding, and the improvement of crops and horticultural varieties, as well as in the conservation of rare and endangered plant species (Nie et al. 2012).

Many species in genus *Acer* L. are important woody plants in forest ecosystems, and trees of this genus are an important component of the deciduous forests of the northern temperate zone (Areces-Berazain et al. 2020). On top of their ecological importance, various *Acer* species have ornamental, economic or medicinal value, and many species are widely cultivated in cities, parks, gardens and other landscaping areas.

Fang et al. (1981) included *Acer pubipetiolatum* Hu et Cheng var. *pingpienense* Fang et W. K. Huin in Section *Integrifolia* in the Flora Reipublicae Popularis Sinicae. Section *Intefrifolia* was subsequently merged with Section *Palmata* in the Flora of China (Xu et al. 2008).

In this study, we report the complete sequence of the *A. pubipetiolatum* var. *pingpiense* cp genome, as well as the phylogenetic position of *A. pubipetiolatum* var. *pingpiense* within the genus *Acer* based on cp genome sequences.

Fresh leaves from *A. pubipetiolatum* var. *pingpienense* were collected from the living individuals cultivated in Kunming Botanical Garden, Kunming Institute of Botany, CAS (102°44′35.19″ E, 25°8′21.50″ N). A voucher specimen (S21C0089) was deposited in Kunming Botanical Garden, Kunming Institute of Botany, CAS (Detuan Liu, liudetuan@mail.kib.ac.cn).

Total genomic DNA was isolated from fresh leaves using the Plant Genomic DNA Kit DP305 (TIANGEN, Beijing, China). A DNAseq library was constructed using the DNAseq Library Prep Kit and genome sequencing was performed using a PE150 method on an Illumina Novaseq platform (Kaitai bio, Hangzhou, China). GetOrganelle v.1.7.5 (Jin et al. 2020) and Fast-Plast v.1.2.8 (https://github.com/mrmckain/Fast-Plast) were used to assemble the genome, and Bandage (Wick et al. 2015) was used to visualize and assess the final assembly. The assembled cp genome of *A. pubipetiolatum* var. *pingpienense* was then annotated using the web-based program Dual OrganellarGenoMe Annotator (DOGMA) with the default parameters (Lohse et al. 2007). The annotated assembly was then carefully checked manually, paying particular attention to start/stop codons and intron/exon boundaries, with the *A. griseum* cp genome (NC_034346) as a reference.

We found that the complete *A. pubipetiolatum* var. *pingpienense* cp genome has a total length of 156,730 bp and a GC content of 37.92%, and comprises an LSC (71,582 bp), an SSC (18,092 bp) and a pair of IR regions (26,743 bp). There are 129 unique genes, including 84 protein-coding genes, 37 tRNAs and 8 rRNAs. The complete *A. pubipetiolatum* var. *pingpienense* cp genome was submitted to GenBank and deposited in the database under accession number MZ944848.

The phylogenetic relationships between *A. pubipetiolatum* var. *pingpienense* and 46 other *Acer* species were reconstructed using a maximum-likelihood (ML) method based on cp genomes downloaded from the NCBI GenBank database, and with *Dipteronia sinensis* (MK_840872) as an outgroup. The cp genomes were aligned using MAFFT v. 7.471 (Katoh and Standley 2013). The alignment was trimmed and used to construct a maximum likelihood phylogenetic tree in the software RAxML v.7.2.8 (Kozlov et al. 2019) using ‘GTR + GAMMA + I’ model, 1,000 bootstrap replicates and the ‘Rapid bootstrapping and search for best-scoring ML tree’ algorithm.

In the resulting phylogenetic tree (Figure 1), *A. pubipetiolatum* var. *pingpienense* has a close relationship with *A. laevigatum*, and these two species fall into section *Palmata* of the Flora of China (Xu et al. 2008, see Figure 1). Our results also suggest that *A. yangbiense* is closely related to *A. leipoense*, and that these two species further cluster together with *A. cesium. A. cesium* is the only member of section *Acer* found in China, although *A. yangbiense* and *A. leipoense* were both placed in section *Lithocarpa* in the Flora of China (Xu et al. 2008). *A. yangbiense, A. leipoense* and *A. cesium* are morphologically very similar, and our results confirm the those of previous research in that section *Acer* should be expanded to include *A. yangbiense* (Li et al. 2019).

**Figure 1.**
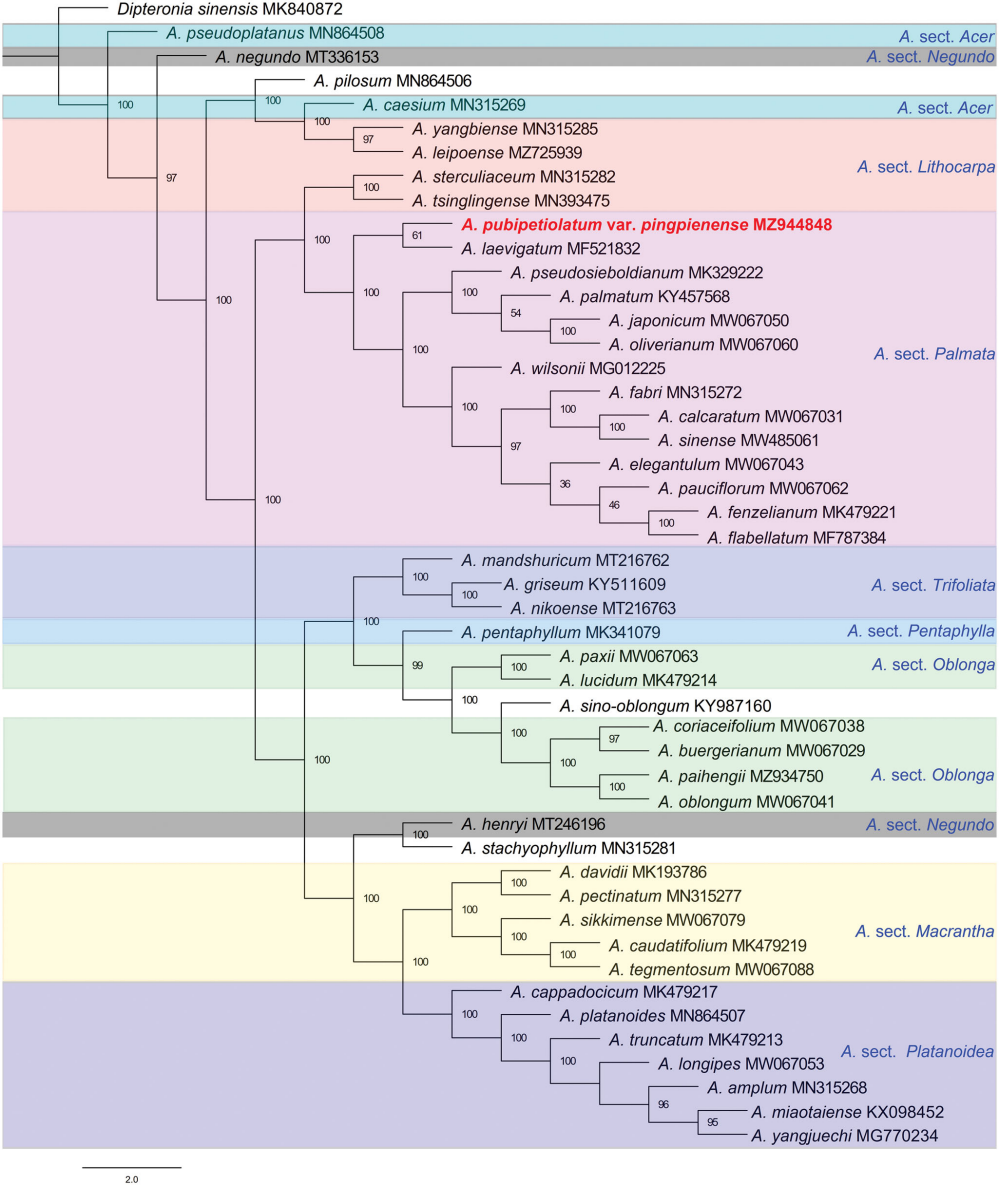
Maximum-likelihood phylogenetic tree. Bootstrap support values are indicated at each node (N = 1,000). Scale bar indicates phylogenetic distance in substitutions per site. The colored boxes represent the sections based on the Flora of China.

Two members of Section *Negundo*, *A. negundo* and *A. henryi*, are found on very different branches of the ML tree. *A. sino-oblongum*, another species included in Section *Palmata*, groups with Section *Oblonga* of the Flora of China (Xu et al. 2008) in our study, and is found in a cluster together with *A. paxii, A. lucidum, A. coriaceifolium, A. buergerianum, A. paihengii* and *A. oblongum* in the phylogenetic tree. These seven species were all placed in Section *Oblonga* in the Flora Reipublicae Popularis Sinicae (Fang et al. 1981).

Our results therefore support the phylogenetic positions of most *Acer* species and sections, while *A. negundo, A. henryi, A. sino-oblongum, A. yangbiense, A. cesium* and *A. pilosum* require further morphological and molecular phylogenetic investigation to confirm their placement within the *Acer* phylogenetic tree.

## Data Availability

The chloroplast genome sequence can be accessed via accession number MZ944848 in GenBank (NCBI). The associated BioProject, SRA, and BioSample numbers are PRJNA758035, SRR15672864 and SAMN20990176, respectively.
